# Quantifying temporal isolation: a modelling approach assessing the effect of flowering time differences on crop-to-weed pollen flow in sunflower

**DOI:** 10.1111/eva.12222

**Published:** 2014-12-02

**Authors:** Marie Roumet, Adeline Cayre, Muriel Latreille, Marie-Hélène Muller

**Affiliations:** 1 INRA, UMR 1334, Amélioration Génétique et Adaptation des Plantes méditerranéennes et tropicales (AGAP)Montpellier Cedex 1, France; 2 ETH Zurich, Institute of Integrative Biology (IBZ)Zurich, Switzerland

**Keywords:** crop-to-weed gene flow, paternity analysis, phenology, pollen dispersal, sunflower (*Helianthus annuus*), temporal isolation

## Abstract

Flowering time divergence can be a crucial component of reproductive isolation between sympatric populations, but few studies have quantified its actual contribution to the reduction of gene flow. In this study, we aimed at estimating pollen-mediated gene flow between cultivated sunflower and a weedy conspecific sunflower population growing in the same field and at quantifying, how it is affected by the weeds' flowering time. For that purpose, we extended an existing mating model by including a temporal distance (i.e. flowering time difference between potential parents) effect on mating probabilities. Using phenological and genotypic data gathered on the crop and on a sample of the weedy population and its offspring, we estimated an average hybridization rate of approximately 10%. This rate varied strongly from 30% on average for weeds flowering at the crop flowering peak to 0% when the crop finished flowering and was affected by the local density of weeds. Our result also suggested the occurrence of other factors limiting crop-to-weed gene flow. This level of gene flow and its dependence on flowering time might influence the evolutionary fate of weedy sunflower populations sympatric to their crop relative.

## Introduction

Flowering phenology is highly variable, both within and among plant populations (Hendry and Day 2005; Elzinga et al. 2007). This variation, which is usually due to a combination of environmental and genetic effects, may strongly affect mating patterns: the more the flowering periods of two individuals overlap, the higher is their chance of mating with each other. This phenomenon is known as temporal assortative mating (Weis and Kossler 2004).

Flowering time differences could act as a major barrier to gene flow between sympatric populations, thereby contributing to reproductive isolation. In the context of local adaptation, temporal reproductive barriers may be targets of selection limiting the exchange of potentially maladapted alleles between populations living in contrasting habitats (i.e. the process of reinforcement). Evidence for reinforcement of flowering time differences has been reported in the grass species *Anthoxanthum odoratum*, both between metal-tolerant and nontolerant populations across a mine boundary (Antonovics 2006), and between experimental populations, at the boundaries between plots subject to different fertilizer treatments (Silvertown et al. 2005). The coexistence between a crop and a weedy relative is a system where reinforcement due to flowering time could occur (Ellstrand et al. 2010). Indeed, when cultivated and weedy forms grow in the same location, crop-to-weed gene flow could recurrently bring domesticated alleles (which are known to be detrimental in the wild, Stewart et al. 2003) into the weedy population and then hinder its divergence from the crop. To go deeper into the mechanisms underlying flowering time as a possible target for reinforcement, an important step is to test and quantify its actual contribution to the reduction of gene flow, which has rarely been carried out.

Sunflower (*Helianthus annuus* L.) is an outcrossing, bee-pollinated species, native from North America (Harter et al. 2004). In Europe, weedy sunflowers have been recently reported within sunflower fields (Vischi et al. 2006; Muller et al. 2009). These plants most probably descend from crop-wild hybrids unintentionally introduced through the seed lots (Muller et al. 2011) and exhibit a wide diversity of phenotypes, forming a continuum between typical wild plants (notably characterized by the presence of anthocyanin pigmentation in stem and head, seed dormancy and seed shattering, self-incompatibility, branching) and cultivated morphotypes (Muller et al. 2009). Weedy populations partially overlap in flowering with the crop and exhibit a wide variance in individuals' flowering time (Roumet et al. 2013). The observation of persistent weedy populations raises the intriguing question of whether partial reproductive isolation may have helped them to adapt to their new environment (Ellstrand et al. 2010; Roumet et al. 2013). In a previous study of six weedy populations, we provided evidence for a temporal genetic structure, that is, a variable genetic differentiation between individuals as a function of their differences in phenology. Through this pattern of ‘isolation-by-time’, we inferred that crop-to-weed gene flow occurred, and was limited by divergent phenologies (Roumet et al. 2013), but were not able to quantify precisely the relationship between phenology and gene flow.

To describe the relationships between the phenology of an individual and its probability of being pollinated by an individual from another population, parentage analysis is a promising approach. It consists of comparing the genotypes of a mother plant and its offspring to a pool representative of potential paternal plants, that is pollen donors, to identify the pollen parent or to evaluate the likelihood of each male being the father of the considered offspring (for a review see Jones and Ardren 2003). When information is available on the parents, such as phenotypic traits or spatial location, their effects on hybridization rates can be tested. Using this approach, two studies have related an index of phenological divergence to an estimate of pollen flow between populations, one in hybrid zones of *Eucalyptus aggregata* and *Eucalyptus rubida* (Field et al. 2011) and the other between two varieties of maize in an experimental field (Della Porta et al. 2008). In an integrative way, statistical models have incorporated parental information into the parentage analysis, to jointly test and quantify the respective effects of the major landscape and phenotypic traits on mating probabilities (e.g. Burczyk et al. 2002; Oddou-Muratorio et al. 2005). A temporal component can be integrated in these ‘mating models’ by including a phenological distance between potential parents (Klein et al. 2011). To our knowledge, this has been carried out only twice: in a hybrid zone between two closely related ash species (Gerard et al. 2006) and in a natural population of *Beta vulgaris* (De Cauwer et al. 2012).

Here, we focus on one of the persistent populations described in Roumet et al. (Roumet et al. 2013). Our aims were to characterize contemporary crop-to-weed pollen flow by quantifying (i) crop-weed hybridization rate and (ii) the effect of weeds' flowering time on it. To address these issues, we describe a sample of adult plants and their progenies at molecular and phenological levels. We adapted the mating model developed by Oddou-Muratorio et al. (2005) to incorporate the effect of a variable crop-weed temporal distance on mating probabilities. The results allowed us to quantify the contribution of phenological divergence to reproductive isolation between cultivated and weedy sunflowers and to infer the occurrence of other reproductive barriers. We discuss the consequences for the evolution of weedy populations, and how our study system might contribute to ongoing research questions on reinforcement.

## Materials and methods

### Study system and data collection

#### Studied field and phenological description of adult plants

**Figure 1:**
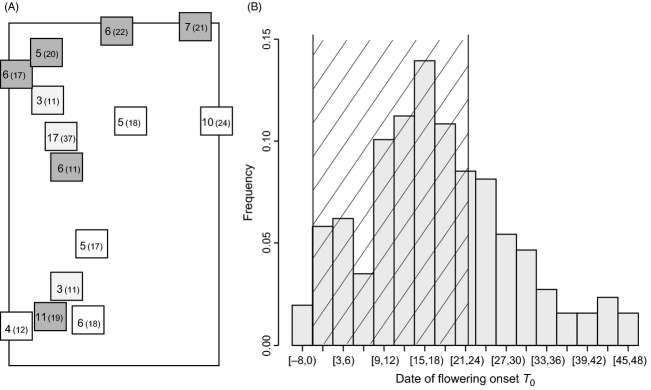
Spatial location and flowering onset of the mother plants sampled in 2009 in the field of Escalquens (2.2 ha). (A) Schematic representation of the field in 2009. Squares represent the quadrats in which weedy plants were surveyed. Their sizes (varying between 1 and 15 m^2^) are not to scale. Numbers in bold type and in brackets correspond respectively to the number of sampled mother plants and to the total number of surveyed weeds in the quadrat. Quadrats are shaded according to the local density of weedy plants: weakly-infested quadrats: white, moderately infested quadrats: light grey and highly-infested quadrats: dark grey. (B) Distribution of the flowering onsets of the sampled mothers. Date 0 corresponds to the start of the crop flowering period (11 July), the dashed zone corresponds to the crop flowering period.

In 2009, the field of Escalquens (2.2 ha) located in Southern France was cultivated with sunflower, with a standard density of 6 crop plants per square metre. It presented large and continuous zones infested with weedy sunflowers, with local densities reaching more than 15 weeds/m² (approx. 0.9 weeds/m² on average). The methods used to sample and describe the phenology of the weedy sunflower population, and the cultivated variety are presented in details in (Roumet et al. 2013). Briefly, a total of 258 weedy plants were followed from the seedling stage to harvest (every 3–7 days between June and August 2009). These plants were chosen by delimiting 14 quadrats over the field and by identifying individually all weeds growing in those quadrats. The quadrats were grouped into three classes according to the local density of weedy plants (i.e. the number of weeds per square metre in the quadrat); weakly, moderately and highly-infested quadrats contained respectively [1, 5], [5, 9] and [9, 25] weeds/m² (Fig.1A). For each weed, we recorded the date of flowering onset, *T*_0_, corresponding to the start of flowering of the primary head. At each visit, we recorded the number of heads flowering on the first-, second-, third-, and fourth-level branches on each weedy plant and estimated the flowering area of the whole sample (i.e. the weeds' flowering area *A*(*t*), (Roumet et al. 2013). Cultivated sunflowers started and ended flowering highly simultaneously; we thus determined a reference period of crop pollen emission by excluding the rare early and late outliers. This ‘crop flowering period’ extended from 11 July to 3 August (Fig.1B).

#### Sexual morph and self-compatibility of weedy adult plants

The sexual morph (hermaphrodite or male-sterile) of each weed was reported. We measured the level of self-compatibility of branched hermaphrodite plants by covering a secondary head with a paper bag before anthesis. Bagged heads were collected just before harvest (8 September), and the number of seeds was counted. Hermaphrodites were scored as self-compatible when at least one seed was produced, as self-incompatible in the absence of seed production and as undetermined when the plant could not be characterized (i.e. unbranched individuals). To account for the methodological problems related to the scoring of self-compatibility (e.g. damaged heads, torn papers bags), an error rate *ε *= 5% was associated to this assignment.

#### Offspring sampling and genotyping

To avoid seed losses due to shattering and bird damages, the primary head of each weedy plant was covered with a paper bag once its flowering finished. Bagged heads were collected just before harvest (8 September). In 2010, 20 seeds per head were sown, for 94 randomly chosen weedy mother plants.

DNA was extracted from 100 mg of fresh leaf sampled at the seedling stage. The genotypes of the offspring were scored at thirteen microsatellite loci: ORS297, ORS337, ORS342, ORS344, ORS371 ORS380, ORS432, ORS610, ORS656, ORS674, ORS788, ORS887 et ORS925 (Tang and Knapp 2003), as described in Muller et al. (2011). The genotypes of the 258 surveyed weeds and of the variety cultivated in 2009 were available for these same 13 loci (Roumet et al. 2013). The theoretical exclusion probability over all thirteen loci was 0.9995 for the 258 adults of 2009 (computed using CERVUS V.3.0.3, Kalinowski et al. 2007).

### Data analysis

#### Paternity analysis: identification of potential crop-pollinated offspring

To assess the extent of crop-to-weed pollen flow, we first used a simple exclusion approach. We used the genotype of the variety cultivated in 2009 and of the weedy mother plants to exclude (or not) the cultivated variety as a father for each offspring (Jones and Ardren 2003). Because genotyping errors, mutations and residual heterogeneity within the variety could contribute to false exclusions, we allowed one mismatch between the variety and its potential offspring. The outcome of this procedure was used to compute, for each sampled mother, the proportion *P*_*c*_ of offspring that could result from pollination by the cultivated variety.

To relate weed phenology to the level of pollination by the crop, we grouped the mother plants into 9 discrete classes according to their date of flowering onset and computed within each class the mean value of *P*_*c*_.

#### Mating model outlook

We assumed that the *oth* offspring of a mother *j* could result from one of three events: (i) self-fertilization with probability *s*_*j*_, (ii) outcrossing with a cultivated plant with probability (1−*s*_*j*_) *π*_*j*,cult_ and (iii) outcrossing with a weedy plant with probability (1−*s*_*j*_) (1−*π*_*j*,cult_). The way *π*_*j*,cult_ and *s*_*j*_ were modelled as functions of the reproductive parameters of the weedy and the cultivated populations, and the way those parameters were estimated are presented in the following.

### Modelling the composition of the pollen clouds

*π*_*j*,cult_ is the probability that an outcrossed offspring of mother *j* resulted from pollination by a cultivated plant. In absence of discrimination between cultivated and weedy pollen during fertilization (e.g. pollen selection or competition), it corresponds to the proportion of cultivated pollen in the pollen cloud fertilizing mother *j*. We modelled it through two components: (i) the total quantities of pollen produced by cultivated and weedy populations and (ii) the proportion of these quantities available at the flowering time of mother *j* (i.e. the temporal distribution of available pollen).

#### Pollen production (male fertility) and its spatial distribution

In the field, the density of cultivated plants was relatively constant; the quantity of cultivated pollen fuelling the pollen cloud surrounding mother *j* was thus assumed to be independent of its spatial location. By contrast, weedy pollen was assumed to be produced either homogenously or as a function of the local density of weedy plants (Fig.1A). In the first case (density independence), the quantity of weedy pollen emitted was modelled using the single parameter *f*_weed_, which represented the weedy male fecundity relative to that of the cultivated plants (*f*_cult_* *= 1 by convention). In the second case (density dependence), weedy male fecundity was allowed to take different values *f*_weed_ (*q*_*j*_), according to *q*_*j*_, the density class of the quadrat where mother *j* was located. *F*_weed_ is the vector containing the values of weedy male fecundities for weakly, moderately and highly-infested quadrats.

#### Temporal distribution of cultivated pollen

The variety cultivated in a given field theoretically consists of a single genotype and starts flowering highly simultaneously. We modelled it as a unique cultivated pollen source emitting at a single date (*T*_cult_, denoted as the crop flowering peak) and described the distribution of the quantity of cultivated pollen through the dispersal kernel *P*_cult_ (*θ*_*d*_*;t*) (eqn 1). Statistically, *P*_cult_ (*θ*_*d*_*;t*) corresponds to the probability for a cultivated pollen grain to participate to the pollen cloud at date *t*; it depends on the temporal distance |*t* − *T*_cult_*|* and is modelled using the family of exponential power functions (eqn 1). Biologically, it combines the variation of crop pollen emission around *T*_cult_, and the delay between the date on which the pollen is emitted and the date on which it reaches the pollen cloud of a weed's head.

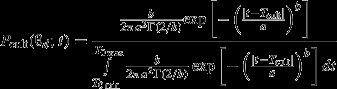
(1)

Г is the classically defined gamma function; *θ*_*d*_ is a vector containing the shape parameter *b* affecting the tail of the dispersal function and the scale parameter *a*, homogeneous to a distance. This function reduces to the bivariate normal distribution for *b *=* *2 and to the bivariate exponential function for *b *=* *1. The denominator allows defining this function on the range of flowering onsets of the sampled mothers [*T*_0 min_, *T*_0 max_].

When the family of exponential power functions is used to model spatial dispersion, eqn 2 gives the mean distance travelled by a pollen grain. In the present case, the computation of *δ* was used to estimate the mean number of days between *T*_cult_ and the flowering date of weedy plants reached by cultivated pollen.

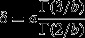
(2)

This model was compared to a null model, where the probability for a cultivated pollen grain to participate to the pollen cloud at date t was assumed to be independent of the value of t and was therefore modelled by a uniform probability function defined on [*T*_0 min_, *T*_0 max_]:

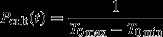
(3)

#### Temporal distribution of weedy pollen

The temporal distribution of weedy pollen (i.e. the probability that a weedy pollen grain participates to the pollen cloud at date *t, P*_weed_ (*t*)) was assumed to be independent of the spatial location within the field. It was modelled as either constant over the period [*T*_0 min_, *T*_0 max_] (eqn 4) or as a function of the weeds' flowering area at date *t* (*A*(*t*), eqn 5).



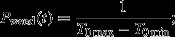
(4)



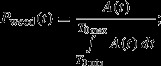
(5)

#### Expected proportion of cultivated pollen in pollen clouds

Finally, the probability *π*_*j*,*cult*_ was computed as the expected proportion of cultivated pollen in the pollen cloud surrounding mother *j*, whose date of flowering onset is *T*_*0j*_, which in the case of density dependence of weedy pollen production and heterogenous distribution over time of both kinds of pollen gives:

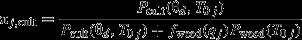
(6)

We wrote similar equations for each combination (eight combinations overall) of alternative hypotheses by replacing the corresponding components by its counterparts: that is using *f*_weed_ for density independence, *P*_cult_ and *P*_weed_ for homogenous distribution of cultivated and weedy pollen over time, respectively.

#### Model for self-fertilization

The propensity *s*_*j*_ of a mother *j* to produce descent by selfing (i.e. the expected proportion of selfed seeds in its offspring) was modelled as:

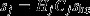
(7)

*s*_hc_ is the selfing rate of self-compatible hermaphrodites. *H*_*j*_ is the probability of a mother *j* being hermaphrodite: *H*_*j*_* *= 0 (resp. *H*_*j*_* *= 1) when *j* was male-sterile (resp. hermaphrodite). When the sexual morph was unknown, *H*_*j*_* *= *f* (*H*_09_)– the frequency of hermaphrodite weeds estimated in the adult population in 2009. *C*_*j*_ is the probability of a mother *j* being self-compatible. *C*_*j*_* *= *1*−*ε* when the plant has been scored as self-compatible, *C*_*j*_* *= *ε* when it has been scored as self-incompatible and *C*_*j*_* *= *f* (*C*_09_) – the frequency of self-compatible hermaphrodites in 2009 – when the self-compatibility was undetermined. *ε* is the error rate associated to the measure of self-compatibility.

### Maximum-likelihood estimation of the pollen dispersal, male fecundity and selfing parameters

#### Log-likelihood function

The probability for offspring *o* to have the multilocus diploid genotype *g*_*o*_ was as follows:

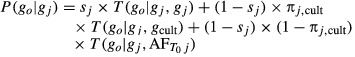
(8)

where, *T*(*g*_*o*_|*g*_*j*_, *g*_*x*_) is the Mendelian segregation probability of the offspring genotype (*g*_*o*_) given the genotype of the mother (*g*_*j*_) and of the father (*g*_*x*_) (Meagher 1986). *g*_*x*_* *= *g*_*j*_, the genotype of the mother, in case of selfing, *g*_*x*_* *= *g*_cult_ in case of outcrossing with the cultivated variety and *g*_*x*_* *= AF_*T0 j*_ – the allelic frequencies in the weedy pollen in the pollen cloud that fertilizes mother *j* – in case of outcrossing with a weedy plant.

No significant spatial genetic structure was detected within the weedy population (data not shown). We thus assumed no spatial variation in the genetic composition of the weedy pollen cloud. The frequencies *AF*_*T0 j*_ were computed under two alternative strategies: first, we computed it from the genotypes of the 258 weedy plants surveyed in 2009; second, we considered that the temporal genetic structure of the adult population (for details see Roumet et al. 2013) influenced the genetic composition of the pollen cloud and computed the frequencies AF_*T0 j*_ from the genotypes of the sampled individuals which were in bloom the interval [*T*_*0 j,*_ *T*_*0 j*_+15] (i.e. an upper limit of the flowering period of the collected head). Due to the incomplete sampling of the adult population, it happened that some rare alleles observed in the offspring of mother *j*, were absent from the genotype of the *n*(*T*_*0j*_) individuals used to computed the allelic frequencies AF_*T0 j*_. To limit the impact of this bias on the likelihood function (eqn 9), the allelic frequencies AF_*T0 j*_ were corrected by adding these new alleles at a frequency of 1/2 *n*(*T*_*0 j*_).

The log-likelihood function of all observed progenies was given by the sum of the log-likelihood of the *o* offspring.

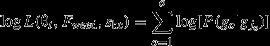
(9)

#### Fits, estimation, confidence interval and model selection

The differences between the models used to describe crop-to-weed pollen flow come from (i) the functions used to model the temporal distribution of cultivated and weedy pollen, (ii) the inclusion (or not) of local variations of weedy male fecundity as a function of weedy plant local density and (iii) the consideration (or not) of temporal variation in the computation of the allelic frequencies in the weedy pollen cloud.

For each model, fits were achieved by maximizing the log-likelihood function (eqn  9) with respect to the following parameters: level of selfing (*s*_hc_), crop flowering peak (*T*_cult_), dispersal parameter (*θ*_*d*_) and effect of weeds local density (*F*_weed_). The log-likelihood function was maximized numerically following a quasi-Newton algorithm (using the built-in R function optim, R Development Core Team 2009).

For the models that were nested into each other (e.g. normal within exponential power dispersal kernel, and density-independent within density-dependent weedy male fertility), we used a likelihood-ratio test (LRT) to test whether the more complete model achieved a significantly better fit: the deviance (i.e. twice the difference of log-likelihood between the complete model and the nested model) was compared to a chi-squared distribution with a number of degrees of freedom equal to the difference in the number of parameters between the complete model and the nested model.

Confidence intervals for the parameters were computed by performing 1000 bootstrap replications, using families as sampling units. Bootstrapped data sets were built to contain the same total number of offspring as in the real data set (for details see De Cauwer et al. 2012).

#### Goodness of fit

Using the predicted values of *π*_*j,*cult_ and *s*_hc_, we computed the expected crop pollination rate of each sampled mother *j* under the best fitted model. Results were compared to the outcome of the exclusion procedure (i.e. the proportion of potential crop-pollinated offspring *P*_*c*_ and its variation across time).

## Results

### Facts about the parental population

Mother plants were spread over the field (Fig.1A). The weedy population, as well as the studied weedy mother plants, included a vast majority of hermaphrodites that were mainly self-incompatible (i.e. the population and the sample of mother plants respectively contained 92% and 90% of hermaphrodites plants of which 82% and 73% were self-incompatible; for details see electronic supplementary material Table S1). Their dates of flowering onset were distributed from *T*_0 min_* *= 10 July to *T*_0 max_* *= 22 August; only one plant started flowering before the beginning of the crop flowering period and approx. 23% flowered after the end of the crop flowering period (Fig.1B).

### Identification of potential crop-pollinated offspring

**Figure 2:**
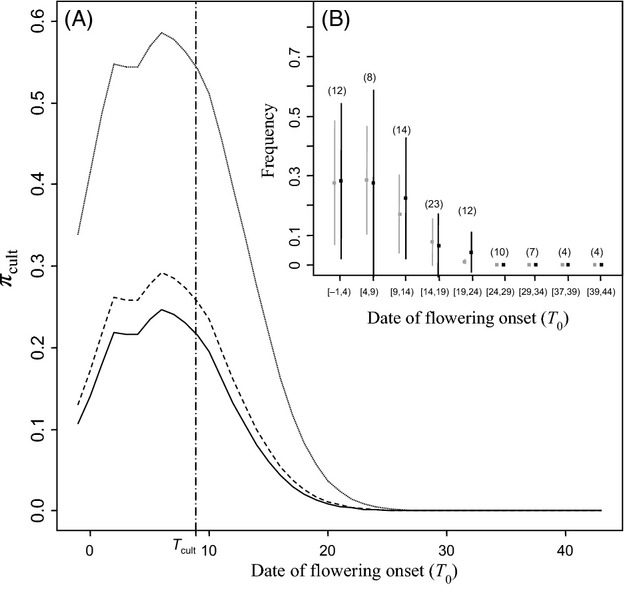
Temporal variation of crop-to-weed pollen flow (A) Expected proportions of crop-pollinated offspring in the outcrossed progeny of weedy plants located in weakly (dotted line), moderately (dashed line) and highly (solid line) infested quadrats. All predictions were obtained under the best model. (B) Mean prediction (±SE) of crop pollination rate in the offspring of sampled mothers plants grouped into 9 phenological classes. Predictions were either obtained with an exclusion procedure (*P*_*c*_, grey bars), or using parameter estimations yielded by the best model (black bar). Numbers in parentheses correspond to the number of mother plants in each phenological group. The vertical line corresponds the estimate of the date of the crop flowering peak: *T*_cult_.

Among the 1656 genotyped offspring, 227 (13.7%) could result from crop pollination. This number was slightly lower when no mismatch was allowed for the exclusion procedure (197, 11.8%). The proportion *P*_*c*_ of crop-pollinated offspring per mother plant varied from 95% to 0% and decreased across time: on average, *P*_*c*_ was equal to 28% in the earlier-flowering group of mother and decreased to 0% in the later-flowering ones. Within flowering groups, the range and the standard deviation of *P*_*c*_ were high and tended to decrease across time (Fig.2B).

### Mating model

#### Model selection

With regard to the temporal dispersion of cultivated pollen, we found that the normal dispersal kernel provided fits similar to the exponential power kernel and better than the exponential kernel (Table1). These fits were clearly better than those obtained under the null model (i.e. uniform probability distribution, eqn 3; *P* value <10^−30^ for all likelihood-ratio tests over the different models).

**Table 1:** Quality of the fit of models with different combinations of alternative hypotheses, evaluated through *L*, the log-likelihood of the data set. In the different models, the dispersal of cultivated pollen was described using either a constant density function (null model) or the family of exponential power functions (exponential, normal, or exponential power function); the temporal distribution of weedy pollen was described by a constant function or as proportional to the weeds flowering area (dispersal of weedy pollen: cst or var, respectively); an effect of local density on weedy male fecundity was included (yes) or not (no), and allelic frequencies in the pollen cloud were computed as either constant or variable over the season (AF: cst or var, respectively). The *L* value of the best model (see text) is highlighted in bold

			Dispersal of weedy pollen: cst		Dispersal of weedy pollen: var	
Temporal distribution of cultivated pollen	Effect of local weed density	d.f.	AF: cst	AF: var	AF: cst	AF: var
			−L	−L	−L	−L
Null	No	3	34941.2	36695.7	34892.9	36647.2
Exponential	No	4	34825.4	36579.7	34818.6	36649.2
Normal	No	4	34819.8	36574.2	34817.8	36572.2
Exp.power	No	5	34819.8	36574.2	34817.3	36571.6
Null	Yes	5	34917.8	36672.4	34864.6	36619
Exponential	Yes	6	34793	36547.9	34790.6	36623.5
Normal	Yes	6	34788.8	36543.4	34787	36541.5
Exp.power	Yes	7	34788.6	36543.1	34786.9	36541.5

We found a significant effect of the weeds' flowering area and of the local weed density (Table1). Indeed, best fits were obtained when the temporal distribution of weedy pollen was modelled as a function of the temporal variation of the weeds' flowering area (eqn 5) and when the quantity of weedy pollen was allowed to take different values in weakly, moderately and highly-infested quadrats (*P* values were <10^−10^ for all likelihood-ratio tests over the different models). By contrast, we didn't detect significant effect of the temporal genetic structure of weedy adult population: models in which we assumed no temporal variation in allelic frequencies provided better fits.

To summarize, the model that was best supported by our data (referred to as the ‘best model’ in the following) modelled the dispersion of cultivated and weedy pollen by a normal dispersal kernel, assumed that the quantity of weedy pollen varied both temporally (as a function of the weeds' flowering area) and spatially (as a function of weeds local density) and assumed no temporal variation of the genetic composition of the weedy pollen cloud.

#### Parameters values

Under the ‘best model’, the selfing rate of self-compatible hermaphrodites was estimated to *s*_hc_* *= 9.7% with a 95% confidence interval (CI) of 3.8–16.9. The estimate of the date of the crop flowering peak *T*_cult_ was 8.84 (CI 6.83–10.63), corresponding to 19 July. As expected, this date falls in the time period on which we observed that the cultivated field was fully blooming. The other outcomes of the model are detailed below.

#### Dispersal kernel of cultivated pollen

The normal dispersal kernel obtained under the ‘best model’ implies that the probability of crop-to-weed pollen flow rapidly dropped as the temporal distance between weedy and cultivated plants increased (Fig. S1). For a cultivated pollen grain, the probability to participate to the pollen cloud of weedy plants that flowered after the end of the crop flowering period (*i.e*. separated from *T*_cult_ from at least 14 days) was <10^−2^. On average, there was an offset of *δ *= 6.25 days (CI 4.85–9.04) between the crop flowering peak *T*_cult_ and the flowering date of weedy plants that were reached by this pollen. The scale parameter *a* was estimated to 7.05 (CI 5.47–10.20).

#### Weedy male fertility

The weedy male fertilities were estimated to 4.59 (CI 2.19–8.18), 15.7 (CI 8.19–34.16) and 19.81 (CI 11.45–37.96) in the weakly-, moderated- and highly-infested quadrats respectively. These values were always higher than 1 suggesting that the weedy population produced more pollen than the cultivated variety. They were greater than expected relative to the numbers of weedy and cultivated plants in the quadrats (<0.9, between 0.9 and 1.5, and between 1.5 and 4.1, respectively).

#### Composition of the pollen cloud and mating patterns

Using the values of dispersal and fecundity parameters estimated under the best model, we computed the expected proportion of crop pollination in the outcrossed progeny of a mother plant, as a function of its flowering onset and of the local weed density in the quadrat where it was located (*π*_,cult_, Fig.2A). This proportion was predicted to be maximum for mother plants that flowered at the date *T*_cult_ in weakly infested quadrats. Maximum proportions yielded by the model reached 58%, 29.2% and 24.6% in weakly, moderately and highly-infested quadrats, respectively. They rapidly decreased to 0% in the offspring of late-flowering weeds.

### Goodness of fit

The mean predicted crop pollination rate of sampled mothers was 10.4%. It decreased from approx. 30% in the earlier flowering groups of mothers to 0% in the latest ones (Fig.2B). Such results were highly consistent with the outcome of the exclusion procedure. Indeed, the observed frequency of offspring that could descend from the cultivated variety (Pc) was equal to 11.4% on average and decreased across time from approx. 30% to 0%. High levels of similarity among predicted hybridization rates and the observed frequency of crop-compatible offspring were observed within the nine precocity groups (Fig.2B).

## Discussion

The objective of our study was to quantify pollen-mediated gene flow between cultivated sunflower and its sympatric weedy relative, and to assess its dependence on the weeds' flowering time. For that purpose, we adapted a spatially-explicit mating model (Oddou-Muratorio et al. 2005), replacing physical distance by temporal distance between the crop and each weedy plant. Below, we discuss the validity of our approach, as well as the biological and evolutionary implications of our results.

### Features, benefits and potential for improvements of the modelling approach

The nature and attributes (i.e. relative contribution to reproductive isolation, genetic basis) of the various kinds of reproductive barriers, as well as their interactions with each other drive the build-up of reproductive isolation between populations and are then great determinants of the divergence and potentially speciation process (for further discussion see Widmer et al. 2009) As highlighted by Lowry et al. (2008), great progresses in the identification of the components of reproductive isolation have recently been made. However, few studies have attempted to quantify their effect. For instance, reproductive barriers have repeatedly been identified within the genus Helianthus and between the cultivated and the wild and weedy forms of *Helianthus annuus* (e.g. Burke et al. 2002aa; Mercer et al. 2006; Sambatti et al. 2012), but the data collected and the methodologies used didn't allow these studies (i) to precisely describe the phenological component of reproductive isolation and (ii) to separate the respective effects of the spatial, prezygotic and postzygotic processes on reproductive isolation. A modelling framework can fill this gap by overcoming the limitation of correlative approaches. Mating models do, however, have practical and methodological disadvantages: they require detailed information on individuals' location and phenotype which are time consuming to collect and use simplifying assumptions.

Here, we estimated a high heterogeneity in hybridization rates between the cultivated variety and the weedy mother plants, and quantified the contribution of phenological (date of flowering onset) and landscape factors (weedy plants density within the neighbourhood) on this variation. Some features of our results support their biological relevance and thus the validity of our approach, despite our assumptions. First, we are in line with results previously obtained and with field observations (Roumet et al. 2013). Indeed, we demonstrated that the crop-weed hybridization rates were maximum during the crop peak flowering period and decreased across the season, along with the increase of crop-weed flowering time divergence (Fig.2A and B). Second, the best-fitting dispersal curve for cultivated pollen dispersion around the date of emission, *T*_cult_, is a normal dispersal kernel (Fig. S1, Table1). Consistent with the fact that mating is usually more limited by time than by space (Hendry and Day 2005), this result constitutes a major difference with the fat-tailed dispersal kernel generally reported for pollen spatial dispersion (Ottewell et al. 2012). Last but not least, our model yielded estimates of crop-weed hybridization rates very close to the hybridization rates estimated by a simple exclusion approach (Fig.2B).

By contrast, we did not detect a significant effect of phenological assortative mating within the weedy population (i.e. no effect of the inclusion of its temporal genetic structure into the model). Although surprising, this negative result may be due to a low precision in the estimation of allele frequencies in the temporal subsets of the data (i.e. allelic frequencies were estimated from <50 individuals in approx. 20% of the data subsamples).

Despite a crop flowering period extending over 19 days, we modelled the temporal pattern of crop pollen emission by a single date: *T*_cult_. This simplification proved useful regarding our objectives but was biologically unrealistic: the temporal distribution of cultivated pollen included in the model (eqn 1) accounts for the combined effects of its temporal dispersal ability (i.e. the probability for a cultivated pollen grain emitted at a given date to reach an ovule at any date) and of the distribution of crop pollen emission around *T*_cult_. Distinguishing these two components requires recording the temporal distribution of the flowering onsets of the cultivated plants and including it into the computation of crop-weed temporal distance (e.g. Field et al. 2011). Such an approach could allow a more accurate description of the pattern of pollen temporal dispersal by itself, for instance its anisotropy (see Austerlitz et al. 2007), the optimal lag between crop pollen emission and reception (e.g. De Cauwer et al. 2012), as well as pollen viability.

### Crop-to-weed pollen flow: temporal isolation and other reproductive barriers

We quantified the average level of crop-weed hybridization rate (i.e. 10.4%) as well as its temporal variation (hybridization rate varies from 30% to 0% for the latest flowering weeds, Fig.2B). These estimates are comparable to the hybridization rate of 27% estimated by Arias and Rieseberg (1994) between adjacent cultivated and wild plots of *H. annuus*. However, they also indicated a relatively low level of crop-to-weed pollen flow considering the respective sizes of the cultivated and weedy compartments. For instance, within the moderately infested quadrats (i.e. where the ratio between the numbers of cultivated and weedy plants was approx. 1:1), the estimated frequency of hybrids in the outcrossed progeny of weedy plants never exceeded 29% (Fig.2A). This suggests that in addition to phenological divergence, crop-weed hybridization rate could be limited by additional factors, acting both before and after pollination.

On the one hand, as demonstrated in the case of maize (Baltazar et al. 2005), a reduction of pollen production could have evolved in the cultivated form as a by-product of domestication and plant breeding. To produce modern varieties of sunflower, breeders have broken the self-incompatibility system and introduced a male-sterile cytoplasmic sterility system (Gandhi et al. 2005). Crop pollen emission could thus be limited both by selfing and by an incomplete restoration of male fertility in the F1-hybrid variety (or a restoration cost, for review see Delph et al. 2007). However, cultivated sunflower shows an outcrossing rate of approx. 60% (Roumet et al. 2012) and its seed production is improved by an increase of pollinator activity (Degrandi-Hoffman and Chambers 2006; Greenleaf and Kremen 2006). This highlights that a significant quantity of pollen is still produced and transferred between cultivated plants. Expectations on the relative quantity of pollen produced by cultivated and weedy plants are thus not clear and require additional data.

On the other hand, crop-to-weed pollen flow may be limited because of mechanisms inducing preferential mating between individuals of the same type. This has notably been demonstrated in the Chicory crop-wild complex (Hauser et al. 2012). Preferential mating between sunflower weedy plants could result from pollinator behaviour. Studies within sunflower fields have demonstrated that the foraging behaviour of pollinators can be influenced by the sexual morph and genotype of plants (Pham-Delegue et al. 1990; Greenleaf and Kremen 2006). Within weedy populations, there was a wide variation in morphology (e.g. height, pigmentation Muller et al. 2009), which may affect the potentiality of pollination by the crop. This component of mating patterns could be assessed by including additional phenological variables into the model, even if care should be taken on the quite high Type 1 error rates for likelihood-ratio tests used in this framework (Klein et al. 2011).

The fertilization success of crop pollen could also be limited by post-pollination mechanisms. Variable pollination success has already been demonstrated at the intraspecific level, between accessions of *Arabidopsis thaliana* (Carlson et al. 2011). However, even if pollen competition has already been demonstrated between the two annual species of sunflowers, *H*. *annuus* and *H. petiolaris* (Rieseberg et al. 1995), it remains to be tested between weedy and cultivated forms of *H. annuus*.

### Evolutionary consequences of crop-to-weed gene flow: 10%, is that weak or strong?

Gene exchange is a major determinant of evolution in crop-weed-wild complexes (Jenczewski et al. 2003). The transfer of cultivated genes to wild and weedy relatives is known to have created new races of weeds, often more aggressive and better adapted to agro-ecosystems (Ellstrand et al. 1999). By quantifying crop-to-weed pollen flow, the present study provides crucial knowledge to assess whether cultivated alleles are likely or not to be permanently incorporated into weedy populations and to discuss how gene flow may affect the evolutionary trajectory of weedy populations.

First, theoretical models showed that alleles contributing to a fitness advantage should be established even for low migration rates, whereas deleterious alleles could be established if gene flow is sufficient to overcome the effect of selection (Haygood et al. 2003). In the light of the development of genetically modified (GM) crops and herbicide-tolerant (GM or not) varieties, this prediction has made scientists redefine what is a low or a high hybridization rate. For example, gene flow was generally considered as negligible in self-pollinating crops such as rice and wheat, but was nevertheless judged as critical for a sustainable herbicide-resistant technology (e.g. Shivrain et al. 2007; Shi et al. 2008). Imidazolinone-herbicide-tolerant varieties of sunflower (non-GM) have recently been put on the market. The estimated crop-weed hybridization rate of 10.4% highlights the high (unavoidable) risk of the transfer of the herbicide-resistance trait into weedy populations.

More generally, traits selected during the domestication process are commonly assumed to reduce fitness in weedy and wild populations (e.g. absence of seed dispersal and dormancy Stewart et al. 2003). Crop-to-weed gene flow might thus negatively affect the adaptive potential of a newly introduced weedy population, especially at the first steps when it is at low density. Here, we demonstrated that the late-flowering part of the weedy population was receiving less gene flow. This raises the question whether a shift in flowering time could evolve in this population as a way to limit gene flow from the crop and further adapt to field conditions. Testing this evolutionary hypothesis would require to gain insights into the selective constraints (e.g. the selective pressures acting on the different phenotypic traits), the interaction between selection and migration (e.g. the fitness of the various classes of crop-weed hybrids) and into the genetic architecture of phenology and of the presumably selected traits. Such data already exist on crop-wild sunflower hybrids (e.g. Baack et al. 2008; Burke et al. 2002b for a genetic analysis of domestication traits; Snow et al. 1998), but have never been collected under normal agronomic field conditions, namely taking the competition with the crop under consideration. As underlined by Neve et al. (2009), weeds are not static units, and weed management practices would greatly benefit from the consideration of evolutionary principles.

## Conclusions

It is widely admitted that a pre-existing level of reproductive isolation, acting at the pre- or postzygotic stages, is necessary for reinforcement to evolve. However, which level of initial reduction of gene flow is sufficient to allow reinforcement, and how various isolating mechanisms contribute remains an open question. Here, we assessed the temporal component of reproductive isolation and evidenced the occurrence of other components. Pursuing such quantitative approach is crucial to complement the numerous theoretical studies investigating under which conditions reinforcement actually occurs in plants. Moreover, reinforcement due to flowering time evolution has never been evidenced in animal-pollinated species, by contrast with wind-pollinated plants [Hopkins 2013]. Whether reinforcement via diverging flowering times or other, more cryptic, traits can or not occur in our bee-pollinated sunflower, can add to the question of the relationships between pollination mode and flowering time evolution.

## Data archiving

Genotype and phenotype data are available from the Dryad Digital Repository: http://doi.org/10.5061/dryad.jq869

## Supporting Information

**Table S1.** Sexual morph and self-compatibility of hermaphrodites in the sample of weeds surveyed in 2009 and in the subsample of mother plants.

**Figure S1.** Dispersal kernels (normal: black lines, exponential: dashed line, and exponential power: dotted line) estimated under the best model.
