# Supplementary material for: Quantifying temporal isolation: a modelling approach assessing the effect of flowering time differences on crop-to-weed pollen flow in sunflower
Source: Evol Appl. 2014 Dec 2;8(1):64–74. doi: 10.1111/eva.12222 (PMC4310582; doi:10.1111/eva.12222)
Supplement: Supplementary file 1 [file eva0008-0064-sd1.pdf]

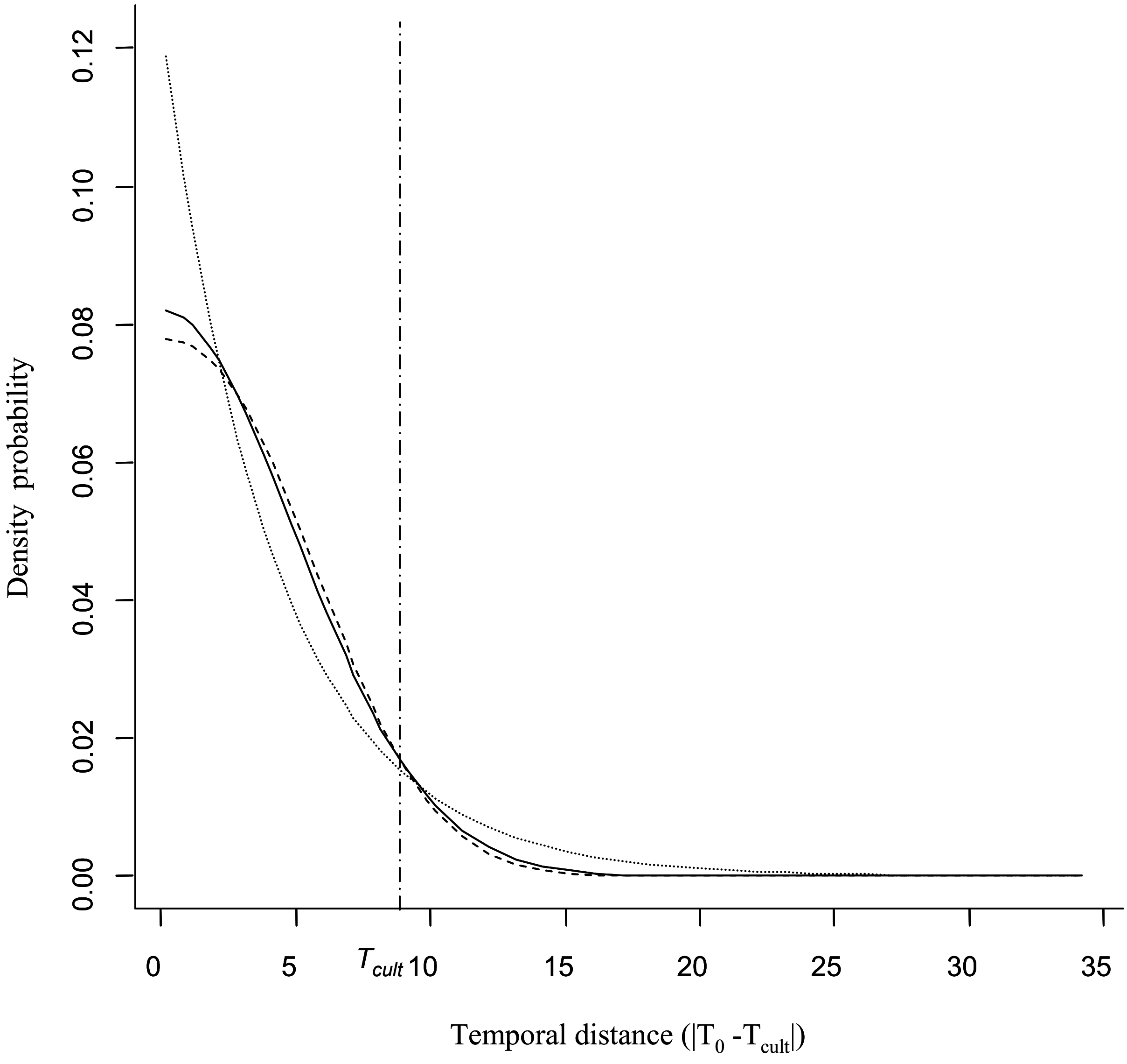

Figure S1: Dispersal kernels (normal: black lines, exponential: dashed line, and exponential power: dotted line) estimated under the best model. The vertical line corresponds to the number of days between T_{cult} and the end of the crop flowering period.
