# Supplementary material for: Quantifying temporal isolation: a modelling approach assessing the effect of flowering time differences on crop-to-weed pollen flow in sunflower
Source: Evol Appl. 2014 Dec 2;8(1):64–74. doi: 10.1111/eva.12222 (PMC4310582; doi:10.1111/eva.12222)
Supplement: Supplementary file 2 [file eva0008-0064-sd2.pdf]

	Sexual Morph			Hermaphrodites' self-compatibility		
	H	MS	NA	SC	SI	NA
Plants surveyed in 2009	235	20	3	90	19	126
Mother plants	84	9	1	35	13	36

Table S1: Sexual morph and self-compatibility of hermaphrodites in the sample of weeds surveyed in 2009 and in the subsample of mother plants. H: hermaphrodite, MS: male-sterile, SC: self-compatible hermaphrodite, SI: self-incompatible hermaphrodite, NA: missing data.
